# Spatially resolved metabolomics and isotope tracing reveal dynamic metabolic responses of dentate granule neurons with acute stimulation

**DOI:** 10.21203/rs.3.rs-2276903/v1

**Published:** 2023-07-25

**Authors:** Anne Miller, Elisa York, Sylwia Stopka, Juan Martínez-François, Md Amin Hossain, Gerard Baquer, Michael Regan, Nathalie Agar, Gary Yellen

**Affiliations:** Harvard Medical School; Harvard Medical School; Brigham and Women’s Hospital; Harvard Medical School; Brigham and Women’s Hospital; Brigham and Women’s Hospital; Harvard Medical School; Brigham and Women’s Hospital; Harvard Medical School

**Keywords:** Brain metabolism, energy metabolism, glucose metabolism, glycolysis, pentose phosphate pathway, inosine metabolism, purine nucleotide phosphorylase, carbon recycling, mass spectrometry imaging, spatial metabolomics, stable isotope tracing, dentate gyrus

## Abstract

Neuronal activity creates an intense energy demand that must be met by rapid metabolic responses. To investigate metabolic adaptations in the neuron-enriched dentate granule cell (DGC) layer within its native tissue environment, we employed murine acute hippocampal brain slices coupled with fast metabolite preservation, followed by mass spectrometry imaging (MALDI-MSI) to generate spatially resolved metabolomics and isotope tracing data. Here we show that membrane depolarization induces broad metabolic changes, including increased glycolytic activity in DGCs. Increased glucose metabolism in response to stimulation is accompanied by mobilization of endogenous inosine into pentose phosphates, via the action of purine nucleotide phosphorylase (PNP). The PNP reaction is an integral part of the neuronal response to stimulation, as inhibiting PNP leaves DGCs energetically impaired during recovery from strong activation. Performing MSI on brain slices bridges the gap between live cell physiology and the deep chemical analysis enabled by mass spectrometry.

## INTRODUCTION

Brain metabolism must constantly adapt to provide the energy needed to support spatially and temporally dynamic neuronal activity. Neurotransmitter recycling and maintenance of ion gradients depend on constant energy supply, and therefore brain function is extremely sensitive to metabolic disruption, as evidenced by cell death following ischemia or seizure activity caused by abnormal blood sugar levels^1–3^. The identification and characterization of metabolic pathways that sustain neuronal function is critical, not only to better understand how neurons regulate their activity and firing rates, but also to provide important insight into disease conditions. Given the importance and clinical implications, and the current lack of understanding of how neurons metabolically support themselves, we sought to elucidate the comprehensive interplay of different metabolic pathways regulated within neurons following acute stimulation.

The methodologies to monitor metabolic processes are expanding rapidly, but studying metabolism on the cellular level comes with challenges, typically requiring the dissection or culture of cells outside of their native tissue environment. The heterogeneity of brain tissue as well as the fast temporal component of neuronal activity has favored live-cell imaging techniques over other biochemical approaches, using autofluorescence signals from a few endogenous metabolites or visualization of individual metabolite species with genetically encoded fluorescent biosensors to gain insight into neuronal metabolism. However, these imaging techniques are currently feasible only for a limited number of molecular species, making it impossible to generate a comprehensive picture of a wide range of metabolic events.

Mass spectrometry (MS), particularly in combination with liquid chromatography (LC-MS), is the favored technique to simultaneously measure many chemical species, as well as to monitor the progress of isotopically labeled fuel molecules through specific metabolic pathways in homogenized tissues or cells. Spatial analysis of metabolism is also possible using mass spectrometry; one approach is matrix-assisted laser desorption/ionization mass spectrometry imaging (MALDI-MSI), in which a laser and matrix are used to desorb and ionize molecules from intact tissue, which are then analyzed by MS. Rather than collecting cells from culture or homogenizing whole tissue, MSI involves the preparation of whole-tissue samples by cryosectioning onto conductive glass slides. Mass spectra are then acquired in a grid pattern throughout the tissue resulting in pixels which are then used to generate and analyze ion images. Originally developed for proteomics and lipidomics applications, recent advances in MSI instrumentation now make it possible to also detect and effectively resolve small metabolites^4,5^. MSI has been successfully applied to describe stationary regional differences in metabolite levels in different tissues, including the brain, and it has the power to quantify isotopically labeled metabolic intermediates^6–9^. The most recent technological advance is the combination of *in vivo* infused stable-isotope tracers with MSI to quantify gross regional differences in metabolic activity as well as isotope tracing in kidney slices^4,10,11^. However, to study the rapid metabolic response to neuronal stimulation, a fast preservation method is needed. We developed an approach that bridges the gap between live-cell imaging techniques with flexible experimental manipulation and temporal precision, and the spatially resolved chemical analysis of MSI. We first perform experiments on acute, *ex vivo* brain slices, and then immediately preserve them by heat inactivation to fix their metabolic state and evaluate the results using MSI. The metabolic experiments can include different forms of stimulation, application of ^13^C-labeled fuel molecules, pharmacological manipulation, and fluorescent imaging.

Using this approach, we have investigated the rapid metabolic response to stimulation of the neuron-enriched dentate granule cell (DGC) layer of the hippocampus and have monitored the incorporation of ^13^C from stable isotope tracers into downstream metabolites. Our results indicate that glucose metabolism becomes faster to meet the increased energy demand introduced by stimulation, but we also reveal some unexpected features that are part of neuronal metabolic response. Using different modes of neuronal activation in hippocampal slices *in situ*, we identify that generation of inosine and subsequent metabolism to ribose 1-phosphate (R1P) by purine nucleotide phosphorylase (PNP) is part of the metabolic response to depolarization in DGCs. The PNP reaction catalyzes the reversible release of R1P from inosine, and some of the R1P can enter the non-oxidative branch of the PPP (non-oxPPP). Using this combination of hippocampal slice preparation and MSI, we thereby not only provide a workflow that allows us to observe the seconds-to-minutes response of energy metabolism to neural stimulation, but we also reveal the role of an underappreciated player in neuronal metabolism.

## RESULTS

### Rapid brain slice preservation for mass spectrometry imaging

**Figure 1:**
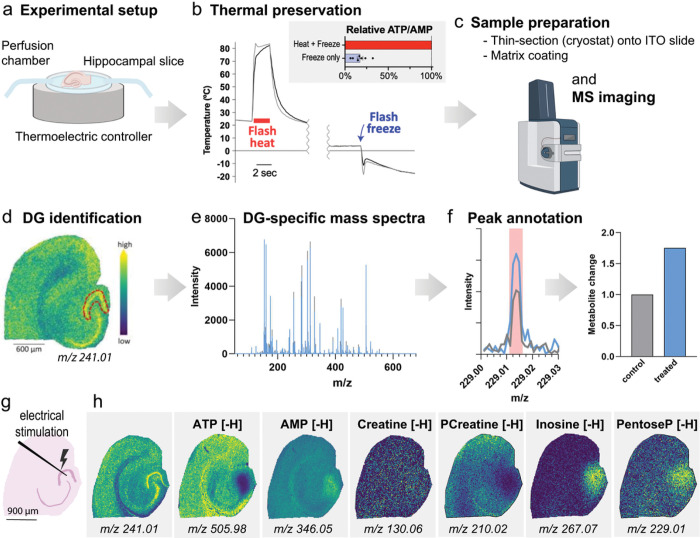
Workflow for acute brain slice experiments, thermal preservation, MALDI-MSI, and data analysis. (**a**) A hippocampal slice was placed in a perfusion chamber in which it can be easily manipulated e.g. chemically stimulated, flushed with stable isotope labeled fuels or treated with pharmacological agents. The chamber bottom is a thermoelectric device that allows immediate metabolic preservation at the desired timepoint. (**b**) Slices were heated to about 80°C for 2 seconds (monitored by an infrared camera) and then cooled to 4°C, while the device pre-chills for subsequent freezing. After flash-freezing, the device with the frozen brain slice was removed and stored at −80°C. Compared to freezing alone, heat-freezing preserves the energy status as indicated by the ATP:AMP ratio. (c) The samples were subsequently prepared for MALDI imaging, which includes cryosectioning onto a conductive (ITO) glass slide and applying matrix. (**d**) After acquisition, the dentate gyrus (DG; red outline) was identified by a feature that reliably outlines neuronal cell body layers (*m/z* 241.01) and (**e**) DG-specific spectra were exported. (**f**) The peaks of the spectra were matched to a library of metabolites of interest and the changes in metabolite levels were compared. Created with BioRender.com. (**g**) Placement of a stimulation electrode in the area of the DG which was stimulated for 30 seconds, in 1-second bursts, and then thermally preserved. (**h**) Ion images of metabolites after electrical stimulation. Pcreatine = phosphocreatine; PentoseP = pentose phosphates.

To combine acute brain slice preparation with MSI, brain slices containing the entorhinal cortex and the hippocampus were submerged in a physiological chamber with a constant flow of oxygenated artificial cerebrospinal fluid (aCSF) (Fig. 1a). This type of experimental setup has been extensively used to study neuronal physiology and allows easy pharmacological manipulation as well as application of isotopically labeled fuels. Metabolic reactions can have extremely fast turnover rates, so robust metabolic quenching is important to ensure accurate metabolite measurements^12,13^. ATP levels are particularly sensitive to sample preparation techniques, as ATP degradation occurs within seconds when samples are not well preserved, accompanied by a simultaneous rise in AMP levels. Heat denaturation rapidly denatures metabolic enzymes and can thereby conserve the metabolic state during subsequent sample preparation steps^12,14^. We accomplished immediate quenching of metabolic reactions with thermoelectric devices that were built into the floor of the physiology chamber (Fig. 1a–b **and Extended Data** Fig. 1a). At the specific time chosen for thermal preservation, the aCSF was removed and slices were flash heated to 80°C for 2 seconds and then cooled to 4°C; soon thereafter the slices were flash-frozen to −20°C (Fig. 1b). We observed that flash heating followed by flash freezing abolished metabolic enzyme activity (**Extended Data** Fig. 1b) and confirmed that the ATP:AMP and phosphocreatine:creatine (PCr:Cr) ratio in brain slices were significantly increased compared with freezing alone, indicating better preservation (Fig. 1b inset and **Extended Data** Fig. 1c). To exclude possible metabolite degradation by heating, we prepared enzyme-free extracts of brain homogenates, which we spotted onto ITO slides and then heated to ~ 80°C for 15 seconds on a hotplate. We then compared the metabolite levels in the heated spots to the levels in the spots of nonheated brain extracts and did not detect any significant changes in metabolite levels induced by the heating procedure (**Extended Data** Fig. 1d). We verified this by similar experiments on yeast extracts (**Extended Data** Fig. 1e and **f**).

For further sample preparation, the brain slices frozen onto the thermoelectric device were stored at −80°C for subsequent thin sectioning in a cryomicrotome (Fig. 1c). The thin sections were mounted onto conductive glass slides (ITO slides) and prepared for MSI. Briefly, the samples were sprayed with matrix that was spiked with ^15^N-labeled standards and analyzed in a timsTOF fleX mass spectrometer with 20 μm spatial resolution, in negative ion mode. The data analysis was focused on the neuron-enriched DG layer, using a region of interest (ROI) drawn based on a feature that highlights the neuronal layers of the hippocampus (*m/z* 241.01, Fig. 1d). The DGC-specific mass spectra (Fig. 1e) were then analyzed by semi-automated peak picking (Fig. 1f) and relative metabolite quantification. To verify that the chosen peak (*m/z* 241.01) can serve as a marker for the neuron-enriched DG layer in the MSI data, we used consecutive cryosections of the same brain slice for MSI, H&E staining and immunofluorescence of the neuronal nuclear marker NeuN and the astrocyte marker GFAP (**Extended Data** Fig. 1g). Overlay of the H&E image, the MSI image, DAPI and immunofluorescence images of the neuronal nuclear marker NeuN and the astrocyte marker GFAP verified that the DG layer mainly consists of neuronal cell bodies, with little contribution of astrocytes (**Extended Data** Fig. 1h).

Lastly, to demonstrate the feasibility of this approach to capture brief regional differences in metabolite levels, we performed a brief electrical stimulation of the DGCs by placing an electrode in the hilus of the DG and stimulated the cells for 30 seconds, in 1-second bursts, and thermally preserved the slice (Fig. 1g). For comparison, we used a hippocampal slice from the same mouse in which an electrode was placed but not stimulated (sham control, **Extended Data** Fig. 1i). Compared to the unstimulated slice, we found that electrical stimulation induced a local adaptation of energy metabolism, including depletion of ATP and phosphocreatine (PCreatine) levels, as well as accumulation of AMP, inosine and pentose phosphates (PentoseP) (Fig. 1h and **Extended Data** Fig. 1j).

### Fast metabolic adaptation of DGCs in response to stimulation

To describe the dynamic metabolic effects of stimulation on DGCs, we applied aCSF containing elevated potassium chloride (KCl, 50 mM) to hippocampal slices. KCl application over a time course of 5 minutes induces uniform and reproducible stimulation in the whole slice, unlike the electrode stimulation, which varies more between trials. We terminated the stimulation with thermal preservation at various time points and analyzed changes in metabolite levels in the DGC layer. MALDI-MSI peak annotation was based on accurate mass, tandem MS measurements, and ion mobility measurements, as detailed in the Methods and **Supplementary Data Table 1.** Isobaric species were not well differentiated, and we therefore excluded certain metabolites such as hexoseP/inositolP from our analysis^10^.

**Figure 2:**
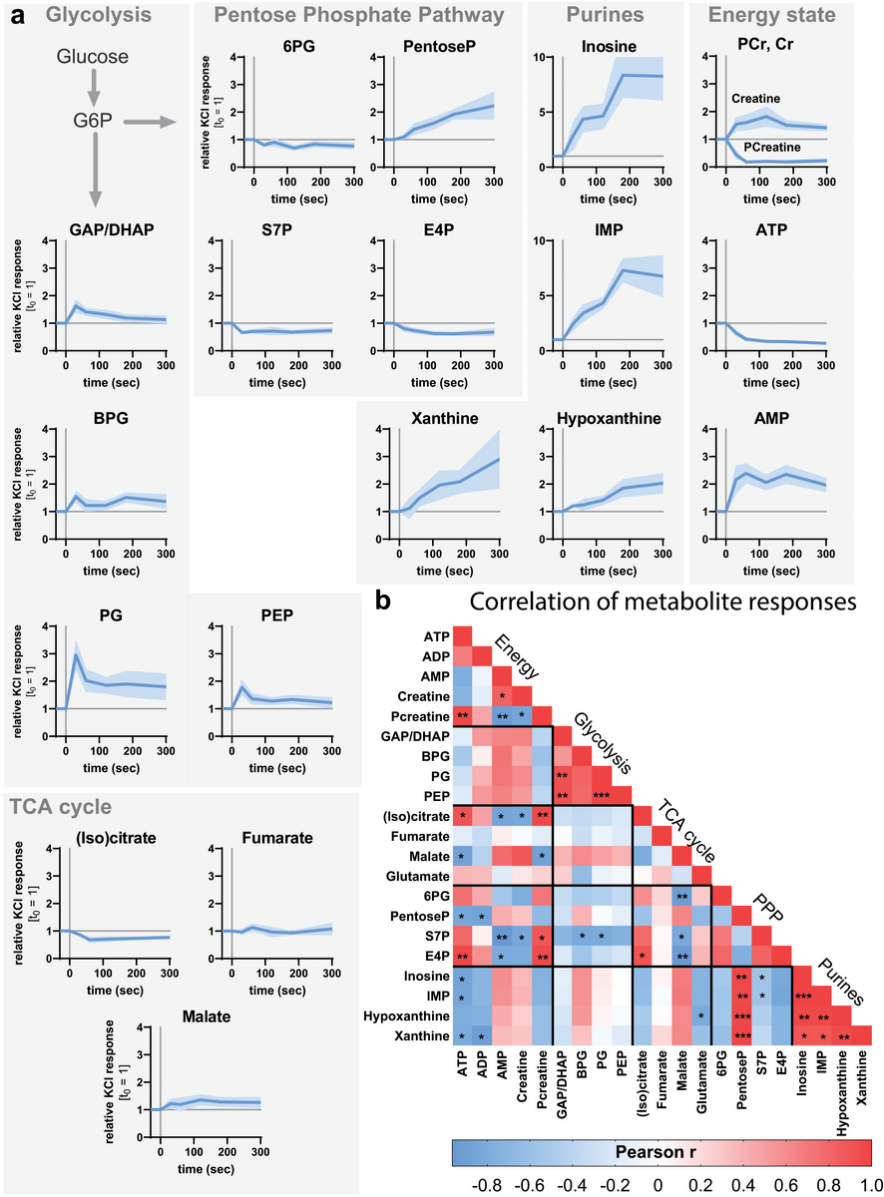
The dynamic metabolic adaptation of the dentate granule cell layer in response to stimulation. (**a**) Hippocampal slices were stimulated with 50 mM KCl for 30 s, 1 min, 2 min, 3 min, or 5 min and metabolite levels were measured in the dentate granule cell layer. (n = 7 mice) All values are mean ±SEM. (**b**) The time-dependent responses of metabolic levels were correlated to each other and the Pearson r was plotted in a heatmap. Values are mean ± SEM; n = 5 – 7 mice. GAP/DHAP = glyceraldehyde 3-phosphate/dihydroxyacetone phosphate; BPG = bisphosphoglycerates, PG = phosphoglycerates; PEP = phosphoenolpyruvate; 6PG = 6-phosphogluconate; PentoseP = pentose phosphates; S7P = sedoheptulose phosphates; E4P = erythrose 4-phosphate; PCr = Phosphocreatine, Cr = Creatine, IMP = inosine monophosphate.

Depolarization induced a strong metabolic demand in DGCs: PCreatine and ATP decreased within the first minute after KCl application, concomitant with an increase in creatine and AMP levels (Fig. 2a and **Extended Data** Fig. 2a). At the same time, glycolytic intermediates increased within 30 seconds of depolarization, but the levels of TCA cycle intermediates and the amino acids glutamate and glutamine remained mostly unchanged (Fig. 2a and **Extended Data** Fig. 2a - **b**). We also detected a linear increase over time in the levels of pentoseP, but other intermediates of the pentose phosphate pathway (PPP) were not changed or were slightly decreased (Fig. 2a and **Extended Data** Fig. 2a). In parallel with changes in adenine nucleotide metabolism, we also detected strongly increased levels of intermediates associated with inosine metabolism, including inosine itself, IMP, and the downstream metabolites hypoxanthine and xanthine (Fig. 2a and **Extended Data** Fig. 2c). To detect possible metabolic co-regulation patterns, we correlated each time-dependent response with every other metabolite and plotted each correlation coefficient (Pearson r) in a heatmap (Fig. 2b). We observed multiple co-regulated metabolic clusters; for instance, the metabolites of energy charge correlated with each other as well as TCA cycle and PPP intermediates. ATP and ADP levels also negatively correlated with intermediates from inosine metabolism. Another cluster was represented by the metabolites of lower glycolysis, which were all positively correlated with each other. Interestingly, rather than correlating with the other intermediates of the PPP, pentose phosphates were strongly correlated with the intermediates of purine metabolism, which were also strongly correlated with each other.

Taken together, the pattern of decreased levels in PCreatine and ATP, together with overall increased levels of lower-glycolytic intermediates, was consistent with the expectation that stimulation induces higher energy demand and elevates glucose metabolism^15–19^(see also **Extended Data** Fig. 2c). While these spatially resolved metabolomics indicate strong regulation of glycolysis and the PPP, metabolite levels alone do not report changes in pathway activity, as increased metabolite levels can arise either from enhanced production rates or from decreased consumption rates. To reveal the changes in pathway activity, we used stable isotope-labeled glucose, [U-^13^C_6_]-glucose, to directly observe the incorporation of the supplied fuel into downstream intermediates.

### Stimulation induces enhanced glucose metabolism in DGCs

**Figure 3:**
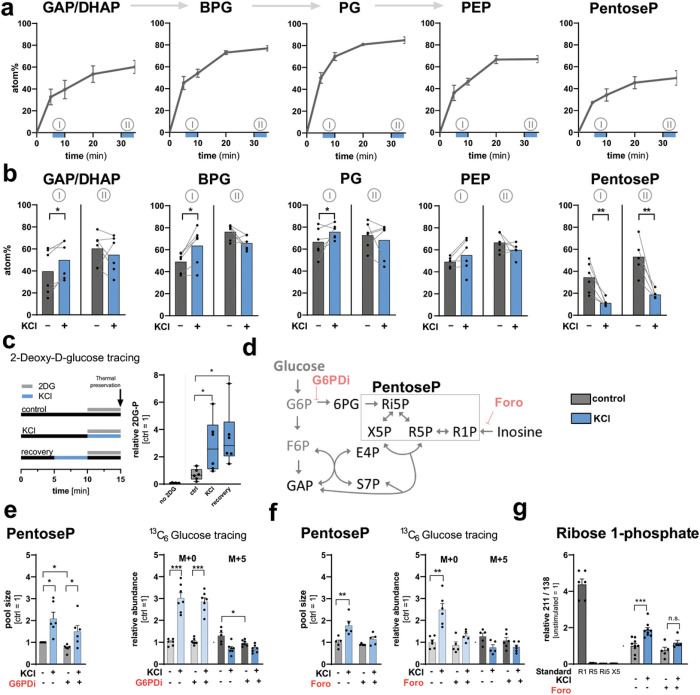
Carbon tracing shows increased glucose metabolism and additional fueling of the PPP by inosine in response to stimulation. (**a**) Hippocampal slices were incubated with [U-^13^C ]-glucose for 5, 10, 20 or 35 min and the average carbon atom labeling (atom%) in the dentate granule (DG) layer was plotted (n = 6 mice). Blue bars under x-axis indicate KCl application periods for non-stationary phase of labeling (I) or after reaching labeling steady-state (II). (**b**) Atom% labeling in control or 5-min-KCl-stimulated DGs after 10 minutes (I) or 35 minutes (II) of label exposure (n = 6 mice). P-values = 0.021, 0.014, 0.050, 0.007, 0.010. (**c**) Hippocampal slices were incubated with 2-Deoxy-D-glucose as indicated and the levels of 2-Deoxy-D-glucose-phosphate were quantified in the DG (n = 6 mice). Whiskers indicate min to max. P-values = 0.042 (bottom bracket) and 0.038 (top bracket). (d) Schematic of the PPP and inosine metabolism as well as the targets of G6PDi and forodesine (Foro). (**e**) The pentoseP levels (left) and labeling from 35 minutes [U-^13^C_6_]-glucose (right) in control and KCl-stimulated DGs in absence or presence of G6PDi (50 μM). (n = 6 mice for metabolomics, n = 7 mice for labeling). P-values = 0.015 (left bottom bracket), 0.02 (right bottom bracket), 0.011 (top bracket) (left graph); from left to right bracket <0,001, <0,001, 0.018 (right graph). (**f**) The pentoseP levels (left) and labeling from 35 minutes [U-^13^C_6_]glucose (right) in control and KCl stimulated DGCs in absence or presence of forodesine (20 μM) (n = 5 mice for metabolomics, n = 6 mice for labeling). P-values = 0.006 (left graph), 0.002 (right graph). (**g**) Contribution of R1P to the total pentoseP pool determined as the ratio of the unique fragment (*m/z*: 211.00) over a shared fragment (*m/z*: 134.91). A two-sided t-test with multiple comparison correction was performed using the two-stage linear step-up procedure with Q = 1%. P-values <0,001 and 0,100. Standards: R1 = ribose 1-phosphate, R5 = ribose 5-phosphate, Ri5 = ribulose 5-phosphate, X5 = xylulose 5-phosphate (n = 9 mice). All values are mean ± SEM, and hypothesis testing was done by two-tailed, paired Student’s t-tests unless otherwise stated. Significance is indicated as p<0.001 (***), p<0.01 (**), or p<0.05 (*). GAP/DHAP = glyceraldehyde 3-phosphate/dihydroxyacetone phosphate; BPG = bisphosphoglycerates, PG = phosphoglycerates; PEP = phosphoenolpyruvate; PentoseP = pentose phosphates; S7P = sedoheptulose phosphates; E4P = erythrose 4-phosphate; PCr = Phosphocreatine, Cr = Creatine.

We first investigated the unstimulated time course of incorporation of ^13^C atoms from [U-^13^C_6_]-glucose into downstream glycolytic metabolites in the DGC layer. We analyzed the main expected isotopologues, which were detected only in tissue incubated with [U-^13^C_6_]-glucose, not in unlabeled control tissue (**Extended Data** Fig. 3a **and b**). Lower-glycolytic intermediates approached an isotopic (pseudo) steady-state level of ^13^C incorporation within 30 minutes, with ~ 60–80% average carbon atom labeling (atom%, mostly as [M + 3]) (Fig. 3a). We then analyzed the effect of membrane depolarization on label incorporation, either during the non-stationary phase of labeling after 5 min of pre-labeling (I) or upon reaching the labeling steady-state after 30 min of pre-labeling (II) with [U-^13^C_6_]-glucose (Fig. 3b and **Extended Data** Fig. 3c). Membrane depolarization changed the kinetics of glucose metabolism, increasing the levels of [M + 3]-labeled glyceraldehyde 3-phosphate / dihydroxyacetone phosphate (GAP/DHAP), bisphosphoglycerates (BPG), phosphoglycerates (PG), and phosphoenolpyruvate (PEP), specifically in the non-stationary labeling phase (Fig. 3b and **Extended Data** Fig. 3c). This was also reflected by increased average carbon atom labeling in the non-stationary phase, indicating that lower glycolysis was accelerated. Using 2-deoxy-D-glucose (2DG) as a proxy for glucose uptake and phosphorylation, we quantified [2DG-phosphate-H] (*m/z*: 243.0275) in the DGC layer and observed higher levels of 2DG-phosphate after depolarization as well as after recovery from depolarization (Fig. 3c), confirming that the initial phases of glucose metabolism were also increased in response to neuronal activation.

### Stimulation induces pentose phosphate formation from inosine

When analyzing unstimulated glucose incorporation into the PPP, we detected ~ 50% average carbon atom labeling of pentoseP from [U-^13^C_6_]-glucose (Fig. 3a). However, labeling of pentoseP was *decreased* by stimulation, in both the non-stationary and the stationary labeling phases (Fig. 3b). Instead, we detected a strong increase in the levels of *unlabeled* pentoseP [M + 0] in response to stimulation (**Extended Data** Fig. 3c). To get insight into the regulation of the PPP during depolarization, we applied an inhibitor for glucose 6-phosphate dehydrogenase (G6PDi), the first and rate-limiting enzyme that controls glucose 6-phosphate entry into the pathway (Fig. 3d)^20^. Whereas G6PDi decreased the levels of 6PG in resting conditions (**Extended Data** Fig. 3d), the stimulation-induced increase of pentoseP levels as well as unlabeled pentoseP [M + 0] (in the presence of [U-^13^C_6_]-glucose) were unchanged (Fig. 3e).

Both the increase in unlabeled pentoseP, rather than labeled pentoseP [M + 5], as well as the lack of response to G6PDi indicated that the strong increase in pentoseP after activation was unlikely to derive from glucose flux into the oxidative branch of the PPP. In addition to the PPP, pentoseP can alternatively be supplied by nucleoside phosphorylases. Purine nucleoside phosphorylase (PNP) breaks down inosine to form ribose 1-phosphate (R1P) and hypoxanthine (Fig. 3d). R1P can be interconverted to ribose 5-phosphate (R5P) by phosphoglucomutase, and hypoxanthine can be further broken down into xanthine. Whereas both R5P and hypoxanthine can then enter the purine salvage pathway via hypoxanthine-guanine phosphoribosyltransferase (HPRT) to generate inosine monophosphate (IMP)^21–23^we did not detect labeling of inosine, IMP or AMP from ^13^C glucose even after 35 min of labeling. We tested if inosine to R1P catalysis is active in DGCs by applying the PNP inhibitor forodesine (20 μM) to hippocampal slices. This inhibitor decreased the levels of pentoseP and inhibited the formation of unlabeled pentoseP [M + 0] in presence of [U-^13^C_6_]-glucose in response to depolarization (Fig. 3f). Of note, inosine accumulated upon incubation with forodesine (**Extended Data** Fig. 3e).

To determine if R1P specifically contributed to the pentoseP pool in response to depolarization, we established a MSI technique to differentiate R1P from other pentoseP species, including R5P, xylulose 5-phosphate (X5P), and ribulose 5-phosphate (Ri5P) using multiple reaction monitoring (MRM). The method used tandem MS to select and fragment a precursor ion [pentoseP-H] (*m/z*: 229.01) with high specificity, and then to monitor the product ions. Because R1P has a slightly different chemical structure than pentoses that are phosphorylated on the C5-oxygen, it generates a unique fragment (*m/z*: 211.00) that can be identified and used for relative quantification (**Extended Data** Fig. 3f **– g**). By using the MRM method, we indeed detected R1P in the hippocampal slices, as defined by the ratio of the unique fragment over a fragment that is shared among all pentose phosphates (*m/z*: 138.91). The level of R1P was increased in response to membrane depolarization, and this increase could be inhibited by the addition of forodesine, confirming increased activity of PNP (Fig. 3g).

Together, our data suggest that whereas glucose metabolism is increased in the DGC layer in response to membrane depolarization, the PPP is additionally fueled by the activity of PNP which breaks down inosine and generates high levels of pentoseP.

### Inosine is generated endogenously in DGCs

To further understand the role of inosine in neuronal metabolism, we aimed to describe the molecular characteristics of the associated pathway, starting with the mechanism of inosine generation. We first considered whether inosine might be imported into neurons from other surrounding cells. Nucleosides can be transported across the cell membrane by two solute carrier (SLC) families, SLC28 and SLC29. Whereas transport by SLC28 family members is sodium dependent and accumulative, the sodium independent SLC29s mediate bidirectional fluxes according to concentration gradients (thus also referred to as equilibrative nucleoside transporters). Analysis of nucleoside transporter expression from published RNA-Seq data indicates that SLC29A1, A2 and A4 are highest expressed in neurons (**Extended Data Fig. 4a**)^24^. SLC29A4 is evolutionarily divergent from SLC29A1–3 and is not considered to be involved in inosine transport, making SLC29A1 and 2 the most likely candidates to transport neuronal inosine^25^.

**Figure 4:**
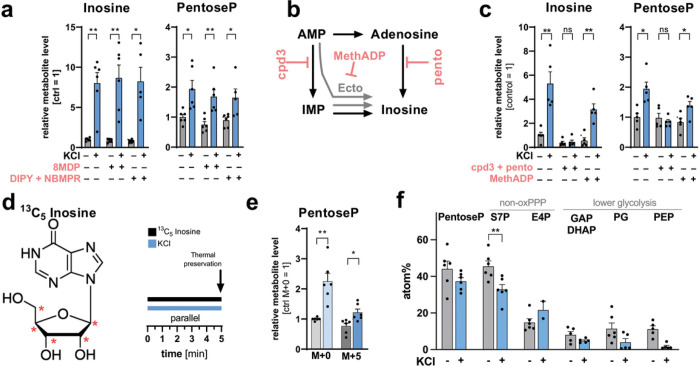
Inosine is generated intracellularly, and exogenously applied inosine can supply carbon atoms to the non-oxPPP and lower glycolysis in DGCs. (**a**) Inosine and pentoseP levels in the DG of control and KCl-stimulated slices in absence or presence of the SLC29A1 and A2 inhibitors 8MPD (1 μM) and DIPY (10 μM) in combination with NBMPR (5 μM) (n = 6 mice). P-values = 0. 003, 0.004, 0.015 (left graph), 0.025, 0.006, 0.025 (right graph). (**b**) Schematic of different inosine precursors. Pentostatin (pento) targets adenosine deaminase, cpd3 targets AMP deaminase, and MethADP targets ecto-5′-nucleotidase (Ecto). (**c**) Inosine and pentoseP levels in the DG of control and KCl stimulated slices in absence and presence of cpd3 + pento or MethADP (20 μM) (n = 5 mice). P-values from left to right brackets = 0.008, 0.124, 0.001 (left graph), 0.024, 0.059, 0.021 (right graph). (**d**) Structure of inosine indicating the^13^C labeling location on the pentose with red stars and schematic of the experimental design for 5 min of inosine tracing. (**e**) The relative change of each isotopologue in pentoseP in control or KCl stimulated DGs. Data were normalized to [M+0] in the unstimulated control (n = 6 mice). P-values = 0.001, 0.029. **f**) Average carbon atom labeling (atom%) of each indicated metabolite after labeling with ^13^C_5_ inosine in control and KCl-stimulated slices (n = 6 mice). P- value = 0.002. All values are mean ± SEM and hypothesis testing was done by two-tailed, paired Student’s t-tests. Significance is indicated as p<0.001 (***), p<0.01 (**), or p<0.05 (*). Data in (**e**) and (**f**) were corrected for natural isotope abundance. GAP/DHAP = glyceraldehyde 3-phosphate/dihydroxyacetone phosphate; PG = phosphoglycerates; PEP = phosphoenolpyruvate; PentoseP = pentose phosphates; S7P = sedoheptulose phosphates; E4P = erythrose 4-phosphate.

To test if inosine is endogenously produced in DGCs themselves, or rather in surrounding cells, we applied different inhibitors for SLC29A1 and A2, namely 2,2’,2’’,2’’’-[[4,8-Bis(hexahydro-1(2H)-azocinyl)pyrimido[5,4-d]pyrimidine-2,6-diyl]dinitrilo]tetrakisethanol (8MDP, 1 μM)^26^ as well as a combination of 2,6-bis(Diethanolamino)-4,8-dipiperidinopyrimido[5,4-d]pyrimidine (DIPY, 10 μM) and 6-S-[(4-Nitrophenyl)methyl]-6-thioinosine (NBMRP, 10 μM)^27^ to hippocampal slices during addition of 100 μM inosine or during depolarization. Either the single addition of 8MDP or the combination of DIPY and NBMPR were able to reduce uptake of exogenous inosine and its conversion to pentoseP (**Extended Data Fig. 4b**). Of note, the inosine level itself still rises with application of exogenous inosine, probably because the inosine that we detect is a combination of intra- and extra-cellular inosine. However, neither inhibitor regime altered the KCl-induced accumulation of inosine and pentoseP (Fig. 4a), suggesting that that the majority of depolarization-induced inosine is generated within the DGCs rather than provided by surrounding cells.

To gain further insight into the regulation of endogenous inosine generation, we aimed to identify the enzymatic framework that could generate inosine in DGCs. Purine metabolism is a non-linear pathway that is highly regulated on each level (**Extended Data** Fig. 2c). Intracellularly, inosine can be directly produced by dephosphorylation of IMP, which is a product of AMP deaminase (AMPD) acting on AMP, or inosine can be directly generated from adenosine via adenosine deaminase (ADA) (Fig. 4b). In addition, extracellular AMP can be converted to adenosine by ecto-5′-nucleotidase (NT5E, also known as CD73) (Fig. 4b). The elevation of inosine and pentoseP levels in response to activation could be abolished by the combined inhibition of AMPD and ADA with cpd3 and pentostatin (20 μM), but not upon single inhibition of either enzyme (Fig. 4c and **Extended Data Fig. 4c**)^28,29^. These results suggest that inosine can be generated from AMP by either of the parallel deaminase pathways. On the other hand, inhibition of NT5E with MethADP (20 μM) did not alter the KCl response (Fig. 4c)^30^, suggesting that the extracellular conversion of AMP to adenosine is not a major source of inosine in DGCs. Taken together, these data suggest that inosine in response to depolarization is mostly generated within DGCs, but that the cells are metabolically flexible regarding the intracellular route of inosine generation.

### Exogenous inosine can fuel glycolysis via the non-oxPPP

Next, to define the potential metabolic fate of inosine in DGCs, we supplied hippocampal slices with [1′,2′,3′,4′,5′−^13^C_5_]-inosine, where only the ribose unit contains carbon-13 as a tracer, thereby allowing us to track its incorporation into primary carbon metabolism (Fig. 4d). We then followed the ^13^C incorporation from inosine into intermediate metabolites of the PPP and lower glycolysis in different non-stationary flux conditions, including a short 5-min incubation with ^13^C inosine and a 5-min pre-incubation with ^13^C inosine (Fig. 4d and **Extended Data Fig. 4d**). In both experimental protocols, we detected rapid incorporation of ^13^C from exogenous inosine into pentoseP: up to 50% of pentoseP could be [M + 5]-labeled from inosine in the resting state, independent of the experimental protocol (Fig. 4e – f and **Extended Data Fig. 4e - f**). Stimulation increased the incorporation of label from exogenous inosine into pentoseP [M + 5], in addition to increasing the production of unlabeled pentoseP [M + 0] (Fig. 4e and **Extended Data Fig. 4f**).

When analyzing downstream metabolites of central carbon metabolism that could derive from inosine, we detected labeling in all intermediates of the non-oxidative branch of the PPP (non-oxPPP) and lower glycolysis, independent of the experimental protocol, indicating that the carbons from the ribose of inosine can propagate into these associated pathways (Fig. 4f and **Extended Data Fig. 4e - i**). When analyzing the specific sedoheptulose-phosphate (S7P) labelling pattern, we detected partially labeled [M + 5] or fully labeled [M + 7] S7P, and lower levels of [M + 2]-labeled S7P (**Extended Data Fig. 4g-h**). This indicates that labeled R1P preferentially entered the non-oxPPP via R5P, which would condense with X5P in the reaction mediated by transketolase (TKT) (indicated in red and blue; **Extended Data Fig. 4h**).

Taken together, these data suggest that inosine is endogenously generated in DGCs in response to stimulation, and that inosine can fuel downstream metabolism.

### Seizure-like events induce inosine-to-R1P catalysis

**Figure 5:**
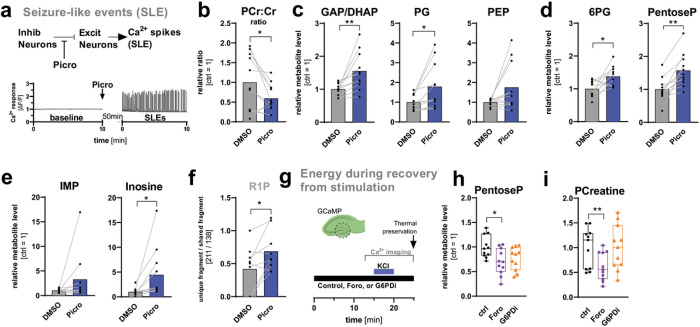
PNP activity is regulated in a model of seizure-like events and helps maintain cellular energy balance when slices are recovering from stimulation. (**a**) Schematic of Seizure-like event (SLE) induction by picrotoxin (Picro). (**b**) Ratio of PCreatine (PCr) /creatine (Cr) and metabolite levels of (**c**) the glycolytic intermediates GAP/DHAP, PG, and PEP in response to 60 min of stimulation with Picro. (**d**) The levels of 6PG and pentoseP as well as (**e**) IMP and inosine in response to 60 min of stimulation with Picro. (**f**) Contribution of R1P to the total pentoseP pool determined with multiple reaction monitoring as the ratio of the unique fragment (*m/z*: 211.00) over a shared fragment (*m/z*: 138.97) in response to 60 min of stimulation with Picro. All values are mean ± SEM (n = 11 mice for each metabolite). P-values from left to right graphs = 0.032, 0.006, 0.015, 0.028, 0.001, 0.039, 0.018. Hypothesis testing was done by two-tailed, paired Student’s t-tests. (**g**) Schematic of the experimental setup to combine live-cell imaging with spatial metabolomics in the DG. Hippocampal slices that are expressing the Ca^2+^ sensor GCaMP were stimulated for 5 min with KCl and then recovered for 5 min while being imaged. (**h**) The levels of pentoseP and (**i**) phosphocreatine (PCreatine) levels in absence or presence of G6PDi (50 μM) or forodesine (Foro, 20 μM) (n = 11 mice for each metabolite). Whiskers indicate min to max. Note that here the control was also KCl stimulated. The statistical analysis for h) and i) was performed using a one-way ANOVA and adjusted with a Dunnett’s multiple comparison correction. P-values = 0.016, 0.002. Significance is indicated as p<0.001 (***), p<0.01 (**), or p<0.05 (*). GAP/DHAP = glyceraldehyde 3-phosphate/dihydroxyacetone phosphate; PG = phosphoglycerates; PEP = phosphoenolpyruvate; 6PG = 6-phosphogluconate; PentoseP = pentose phosphates; R1P = ribose 1-phosphate.

To test if inosine to R1P catalysis in DGCs can also be observed in a different model of neuronal activity, we proceeded to use low dose application of the GABA_A_ receptor antagonist picrotoxin (2.5 μM), which induces electrical activity in neurons^31^. Picrotoxin partially disinhibits the entorhinal cortex - hippocampal circuit and leads to spontaneous seizure-like events (SLEs), creating short Ca^2+^ spikes (Fig. 5a). We were able to detect changes in the energy state of DGCs in response to picrotoxin-elicited SLEs that were similar to those previously seen with KCl stimulation, including a reduced PCreatine:Creatine ratio (Fig. 5b). This was accompanied by increases in the glycolytic intermediates GAP/DHAP, PG, and PEP (Fig. 5c) as well as higher levels of 6PG, pentoseP, IMP, and inosine (Fig. 5d–e). Lastly, we used the previously established MRM to detect the contribution of R1P to the total pentoseP pool and again detected accumulation of R1P levels in response to picrotoxin, indicating engagement of PNP also in this model (Fig. 5f). These data suggest that metabolic responses are conserved between different modes of neuronal activity induction, including increased glycolytic intermediates and engagement of inosine to R1P catalysis in response to KCl stimulation as well as in response to seizure induction in DGCs.

### PNP activity sustains DGC energy balance post-depolarization

Because we observed strong decreases in PCreatine and ATP levels in response to stimulation, we next asked whether PNP activity contributes to restoring cellular energy status after a challenge to the DGC energy reserves (Fig. 2a). The PentoseP generated from the PNP reaction could be used to fuel the PPP (and thence lower glycolysis) or used in the purine salvage pathway to generate or 5-phosphoribosyl-1-pyrophosphate (PRPP). To assess the biological significance for energy recovery of carbon flux via G6PD over carbon flux from inosine, we compared the energy status of the DGC layer after 5-min KCl depolarization plus a 5-min recovery period. We used the fluorescent Ca^2+^ sensor GCaMP6f to monitor neuronal activity and performed thermal preservation and MSI at the end of the recovery period (Fig. 5g). Because both imaging and MSI data derived from the same slice, this enabled correlation analysis between metabolite changes and Ca^2+^ responses (**Extended Data** Fig. 5c-f). Whereas treatment with G6PDi or forodesine did not change the Ca^2+^ response of the slices (**Extended Data** Fig. 5b), inhibition of PNP decreased the overall levels of pentoseP and Pcreatine in DGCs and slightly reduced the ATP levels and increased the AMP levels (Fig. 5h–i **and Extended Data** Fig. 5c - **f**). When correlating the area under the curve (AUC) of the Ca^2+^ response with changes in metabolite levels, the pentoseP levels were decreased in presence of both, G6PDi and Foro (**Extended Data** Fig. 5c). Inhibition of PNP (but not G6PD) slightly increased the levels of AMP, but reduced the levels of ATP and PCreatine (**Extended Data** Fig. 5d - **f**), which can serve as a highly mobile reserve of phosphate [P_i_] to quickly recycle ATP from ADP when energy demands are high (**Extended Data** Fig. 2). Taken together, these data show that whereas G6PDi has no effect on energy state, PNP inhibition induces an energy deficit in DGCs recovering from depolarization.

## DISCUSSION

The combination of acute brain slice preparation with MSI allows us to study transient metabolic changes with spatial and temporal resolution while preserving the brain tissue architecture. This is possible because recent advances in MSI technologies now enable us to gain region-specific information on metabolite levels and stable isotope incorporation into downstream metabolites. While MALDI-MSI can compare regions quantitatively when differences in regional desorption/ionization efficiencies are accounted for^32,33^, here we have not depended on any correction for such differences. Instead, we used MSI to make repeated quantitative measurements on a single anatomically well-defined, neuron-enriched cell layer, in response to different conditions. Effectively, we use MSI as a fine separation tool that is much more precise than any dissection.

One general limitation of MALDI-based MS compared to LC-MS-based approaches is that peak identification is grounded purely on mass spectrometric and tandem MS measurements. LC-MS provides an additional chromatographic separation of isobaric species: for instance, it allows separation of hexoses (C_6_H_12_O_6_) from inositol (also C_6_H_12_O_6_); in brain, this *m/z* peak is dominated by inositol^10^. Because of the difficulty of distinguishing inositol(-phosphates) and hexose(-phosphates) with MALDI-MSI, we chose to exclude the metabolites of upper glycolysis and focused on lower-glycolytic intermediates, relying on phosphorylation of 2DG as a proxy for glucose uptake and phosphorylation (Fig. 3c). Also, some smaller metabolites such as lactate and pyruvate could not be detected reliably with our instrumentation. We were able to use a tandem-MS-based MRM method to distinguish R1P from the other isobaric pentoseP species (Fig. 3g); further separation techniques for MSI approaches, such as additional MRM methods or complementary approaches such as gas-phase ion mobility^34,35^, will be needed to separate more isobaric species to ultimately increase pathway coverage. An additional limitation of MALDI-MSI is the current spatial resolution, which is marginal for obtaining specific signals from single small cells in the brain, which is why we chose to focus on the neuron-enriched DGC layer of the DG (Fig. 1d); it can also be used in other tissues in which groups of similar cells are spatially clustered^10,11^. We anticipate that technological advances will soon enable higher spatial resolution and well-isolated single cell signals^36^.

Our aim was to describe the dynamic metabolic adaptations in response to stimulation in the neuron-enriched DG layer of the hippocampus. The brain is unique in its metabolic requirements as neuronal activation induces local and intense moment-to-moment variations in the metabolic state, mostly to support neurotransmitter recycling and maintain ion gradients. We chose to define the metabolic changes in DGCs in response to two different forms of neuronal activation, one based on a supraphysiological application of KCl and another one based on application of the GABA_A_ receptor antagonist picrotoxin. Whereas electrical stimulation is possible in this experimental setup, the short KCl application has the advantage of depolarizing all cells in the slice uniformly and might challenge neurons in a similar way as observed with spreading depression, which is also referred to as spreading depolarization (SD). These events are characterized by slowly propagating strong changes in transmembrane ion gradients which are followed by periods of synaptic activity loss^37^. SD can occur in patients with brain injury but has also been hypothesized to be the underlying mechanism of the migraine aura and involved in the sudden unexpected death of patients with epilepsy^38,39^. Our second mode of neuronal activity induction, with picrotoxin, works by partially disinhibiting the entorhinal cortex - hippocampal circuit and thereby elicits spontaneous electrical activity in neurons over the time course of an hour. The picrotoxin-induced neuronal activity is characterized by very short activity events, SLEs, that can be compared to events happening during epileptic seizures^31^. With both forms of activation, our results suggest that neuronal activity rapidly increases cellular metabolic demands indicated by changes in the Pcreatine:creatine ratio (Fig. 2a and Fig. 5b). To meet these metabolic demands DGCs increased their glucose metabolism, which is indicated by early accumulation of lower glycolytic metabolite levels (Fig. 2a and Fig. 5c), increased label incorporation from [U-^13^C_6_]-glucose, as well as elevated phosphorylation of 2DG in response to activation with KCl (Fig. 3b–c).

In addition, the rapid increase in glucose metabolism upon stimulation was accompanied by strong mobilization of inosine (by ~ 8-fold; Fig. 2a and Fig. 5e) together with enhanced conversion to pentoseP via the action of PNP (Fig. 4f and Fig. 5f). Similar to our findings, a recent metabolomics-based study found that cognitive stimulation in mice running a Y-maze increased the levels of inosine metabolism associated metabolites in homogenized hippocampi, including inosine itself, hypoxanthine and xanthine and accumulation of inosine levels in response to electroshock seizures has also previously been reported^40,41^.

To understand the role of inosine catabolism in neuronal metabolism, we aimed to define the molecular characteristics of inosine generation in DGCs. Although uptake and metabolism of exogenously supplied inosine is possible in DGCs (Fig. 4e–f and **Extended Data Fig. 4b**), our findings suggest that the inosine is mostly generated intracellularly in response to increased energy demand, rather than imported from surrounding cells, as inhibition of SLC29A1/2 did not alter the KCl-induced increase in inosine levels (Fig. 4a–c). Interestingly, DGCs seem to be metabolically flexible regarding the exact route of endogenous inosine generation, as the single inhibition of AMPD or ADA did not alter the generation of inosine; instead, it required the combined inhibition of both enzymes to diminish inosine generation in response to depolarization (Fig. 4c and **Extended Data Fig. 4c**). The enhanced production of inosine is presumably a byproduct of the higher energy demand and elevated AMP production, as adenylate kinase works to preserve the intracellular ATP:ADP ratio. ATP recycling through various recycling routes is well described in different organs, but the predominant intermediate state varies between tissues. The AMPD-dependent breakdown of ATP to inosine via AMP was previously described in mitochondria-free rat brain extracts^42^. Interestingly, in response to ischemia, the deaminase-mediated degradation of AMP favored generation of inosine via adenosine with little contribution of IMP. In skeletal muscle, vigorous exercise (in humans) or prolonged ischemia of 2 hours (in rats) results in degradation of much of the adenine nucleotide pool to IMP, which was not metabolized further^43,44^. In cardiac muscle, however, degradation of the adenine nucleotide pool occurs via adenosine and inosine, mainly bypassing IMP^45^.

We hypothesize that the specific role of AMP/adenosine deamination and the PNP reaction in DGCs is to provide a source of inosine-derived pentoseP when metabolic demands are high, as inosine catabolism does not depend on ATP (unlike catabolism of glucose, which requires several ATP-dependent phosphorylation steps). Some of the inosine-derived carbons can enter the non-oxPPP and fuel lower glycolysis; blocking the PNP reaction with forodesine can induce an energy deficit when cells are recovering from activation (Fig. 5i **and Extended Data** Fig. 5d - **f**). By consuming phosphate, the PNP reaction, much like the glycogen phosphorylase reaction, may also be capable of buffering the large increases in cytosolic inorganic phosphate [P_i_] that are likely to occur during ATP and phosphocreatine consumption, thus limiting the reduction in the energy of ATP hydrolysis created by phosphate accumulation^46,47^. The preservation of phosphorylation state energy would seem to be the most immediate priority of neuronal metabolism, as it is essential for ion pumping and synaptic function. This energy can come from catabolism of glucose and of PNP-derived ribose, which provide ADP to ATP conversion both through glycolysis and oxidative phosphorylation. The restoration of the total adenine nucleotide pool (which was sacrificed by adenylate kinase and AMP degradation in order to maintain phosphorylation state) is likely to follow afterwards; this can occur either through the nucleotide salvage pathway^21,22^ or through *de novo* synthesis of nucleotides using ribose-phosphate derived either from the PPP or from PNP^48^. Transcellular supply of nucleosides could also play a role in restoration of the adenine nucleotide pool, by uptake of extracellular adenosine produced by ATP or adenosine release from neighboring cells.

Inosine to R1P metabolism via PNP has clear importance in other biological contexts. PNP is established as an essential player in lymphocyte metabolism, and inosine can even completely substitute for glucose in fueling effector T cells *in vitro*^49,50^. On the clinical side, the PNP inhibitor forodesine is currently in a variety of clinical trials as a possible treatment for lymphomas (https://www.clinicaltrials.gov/ct2/results?term=Forodesine&flds=Xabce). The potential significance of inosine catabolism in the central nervous system is also suggested by the phenotype of PNP-deficient patients; although they present with severe immunological disorders, a majority of patients additionally suffer from neurological dysfunction including seizures, developmental delay, and intellectual disability^51–53^. The general importance of purine metabolism in the brain is additionally emphasized by other diseases that are associated with abnormal purine metabolism such as HPRT deficiency (Lesch-Nyhan syndrome) and ADA deficiency. Fully understanding the specific roles and regulatory mechanism of PNP in the brain might present therapeutic options of targeting these pathways in the future. Also, the potential beneficial effects of inosine supplementation for treatment of various central nervous system disorders have been studied since the 1970s and are well reviewed^54–57^.

In summary, using the combination of hippocampal slice preparation and MSI, we introduce a technological advance that bridges the gap between live-cell imaging experiments, which have high spatial and temporal resolution but a very limited chemical dimension, and deep chemical analysis by mass spectrometry. We were able to describe dynamic metabolic adaptations in response to membrane depolarization in DGCs and reveal that elevated glucose metabolism is accompanied by regulation of inosine metabolism. Similar to the extensive work on the role of PNP in immune cells, which enabled the development of forodesine as a therapeutic option, further work will be necessary to fully understand the role of PNP and inosine catabolism in neuronal function and dysfunction, as well as the potential for using inosine as a corrective for metabolic deficits.

## METHODS

### Mice

Both male and female wild-type C57BL/6N mice between P14 and P20 were used. Animals were bred in-house in ventilated cages within a barrier facility, which maintained 12 hr light/dark cycle, regulated cage temperature (24°C) and humidity (53%) and provided ad libitum access to water and food (Picolab Rodent Diet 5053).

All experiments were performed in compliance with the NIH Guide for the Care and Use of Laboratory Animals and Animal Welfare Act. Specific protocols were approved by the Harvard Medical Area Standing Committee on Animals; institutional animal welfare assurance number: D16–00270 (A3431–01); protocol number: IS00001113–6.

### Acute hippocampal slice preparation

On the day of an experiment, a mouse was anesthetized by isoflurane inhalation and decapitated. The brain was immediately extracted, and the cerebellum and prefrontal cortex anterior to the optic chiasm were removed. The hippocampus and remaining tissue were submerged in ice-cold slicing solution: 87 mM NaCl, 2.5 mM KCl, 1.25 mM NaH_2_PO_4_, 25 mM NaHCO_3_, 7 mM MgCl_2_, 0.5 mM CaCl_2_, 25 mM D-glucose, and 75 mM sucrose (osmolality ~ 310 mmol/kg; pH ~ 7.4). The tissue was then glued at the dorsal surface onto the stage of a vibrating Compresstome (Precisionary Instruments), and embedded in 2% agarose. Transverse hippocampal slices were cut at 450 μm thickness into cold slicing solution. Each slice was moved with a glass transfer pipette into 37°C artificial cerebrospinal fluid (aCSF): 120 mM NaCl, 2.5 mM KCl, 1 mM NaH_2_PO_4_, 26 mM NaHCO_3_, 1 mM MgCl_2_, 2 mM CaCl_2_, 10 mM D-glucose (~ 300 mmol/kg, pH 7.4). the osmolarity of all solutions was frequently checked with an osmometer (Wescor; VAPRO 5520). After 30 minutes of recovery, slices were stored at room temperature for 0 to 3 hours before use. All solutions were continuously bubbled with 95% O_2_ and 5% CO_2_.

### Hippocampal slice perfusion and stimulation

All solutions were kept at 38°C in a water bath, and slices were maintained at 34°C throughout the experiment using an in-line heater (Warner TC-324B) and the warmed solutions. All solutions were continuously bubbled with 95% O_2_ and 5% CO_2_.

To perform a physiological experiment, the acute slice was placed in a physiological chamber, in which the bottom is formed by a thermoelectric device (see section on fast thermal preservation).

In all experiments, a baseline period of 5 minutes in control aCSF was used to allow slices to equilibrate. To perform KCl stimulation, the perfusion solution was then changed to 50 mM KCl aCSF (with reduced NaCl to 72.5 mM for osmotic balance) for the indicated time period as indicated in the text. The stimulation time was started when high KCl solution reached the physiological chamber. Perfusion was stopped 15–20 seconds prior to the desired endpoint of stimulation to allow time for solution removal and rapid heat denaturation. For the recovery experiments, the 50 mM KCl solution was changed back to regular aCSF for 5 min before heat denaturation.

### Electrical stimulation

Acute hippocampal slices were moved to a physiological chamber with TEC base and continuously perfused at 5 ml/min with oxygenated aCSF and kept at 34°C. Dentate granule neurons were stimulated antidromically with a concentric bipolar electrode (FHC, Bowdoin, ME) placed in the hilus. Stimulation consisted of 15 repetitions of a 1 sec excitation and 1 sec rest interval (total 30 s activation protocol). Excitation consisted of 1 mA pulses at a frequency of 50 Hz, using an A360 stimulus isolation unit (WPI, Sarasota, FL), controlled by MATLAB software. Following the stimulation protocol, slices were immediately thermally preserved (within 30 seconds after last stimulation pulse).

### Stable isotope and 2DG tracing

For ^13^C_6_ glucose tracing experiments, the glucose in the aCSF was exchanged with an equimolar amount of U-^13^C_6_ glucose (Cambridge Isotope Laboratories) and perfused onto the slice for the indicated time. For the ^13^C_5_ inosine tracing experiments, 1 mM labeled inosine (Omicron Biochemicals) was added to 9 mM unlabeled glucose, to keep the sugar content isocaloric (10 mM in total) in either control aCSF or aCSF with 50 mM KCl. The solutions were applied as indicated in the corresponding figure. For all carbon tracing experiments, a control slice was included in each round of experiments, in which no label was applied.

For 2-Deoxy-D-glucose (2DG) experiments, 1mM 2DG (Millipore Sigma) was added to 9 mM glucose in either control aCSF or aCSF with 50 mM KCl. The solutions were applied as indicated in the figure. A control slice without 2DG was included in each round of experiments.

### Pharmacological manipulations

For pharmacological treatments, drugs were added at their final working volume into either control or KCl aCSF. G6PDi (Excenen PharmaTech) was used at 50 μM with a 20 min preincubation and continued during the KCl stimulation. For the MSI experiments, forodesine (Cayman Chemicals) was used at 20 μM with a 20 min preincubation and continued during the KCl stimulation. 8MDP (Tocris) was used at 1 μM and NBMPR (Tocris) was used at 5 μM in combination with 10 μM DIPY (Tocris) with a 15 min preincubation and continued during the KCl stimulation. Control reactions were carried out in presence of 100 μM unlabeled Inosine (Millipore Sigma). Cpd3 (Millipore Sigma), pentostatin (Selleckchem), and MethADP (Millipore Sigma) were used at 20 μM with a 20 min preincubation and continued during the KCl stimulation. In cases where DMSO was used as a drug solvent, an equivalent volume of DMSO alone was added to aCSF as a vehicle control.

### GCaMP6f expression and Calcium imaging

GCaMP6f^58^ expression in the entorhinal cortex and hippocampus was achieved by intracranial stereotactic injection of P1–2 mouse pups using published methods^59^. Following cryoanesthesia, pups were injected with 200 nl per hemisphere of AAV2/9.CAG.GCaMP6f (Addgene; 100836-AAV9) at 0 μm anterior-posterior, ± 2.0 μm medial-lateral, and − 2.0 μm dorsal-ventral, with respect to lambda.

For Ca^2+^ imaging experiments, acute hippocampal slices were made from mice injected with AAV2/9.CAG.GCaMP6f. Experimental details are further described in Experimental treatments.

Slices were visualized with an Olympus BX51WI upright microscope using either a 4X (NA 0.13) objective or a 10X (NA 0.3) water-immersion objective for experiments that included MSI. GCaMP6f was excited at 480 nm using an AURA III Light Engine (Lumencor, Beaverton, OR), via a multiband dichroic (Semrock Di03-R488/561). Images of emitted fluorescence were filtered (Semrock FF01–512/630) and collected with a cooled-CCD camera (Sensicam QE, PCO) at a rate of 8 Hz. Excitation and image acquisition were controlled with custom-made MATLAB programs (MathWorks, Natick, MA).

To define the Ca^2+^ response, the relative fluorescence change in response to KCl was compared to the baseline before KCl addition, during the maximum of the KCl response and during the recovery. The integration window was adapted between different experiments but kept constant between the treatment groups of each matched experiment. The area under the curve (AUC) calculations were performed in GraphPad Prism, setting the baseline at Y = 1 and ignoring peaks that were 10% of the distance from min to max Y. All peaks had to go above the baseline. Only one peak was identified in each recording and the total peak area was plotted to be correlated with changes in metabolite levels.

### Induction of seizure-like events (SLEs)

SLE experiments were performed essentially as previously published^31^. The main difference was that, instead of a double-perfusion chamber, slices were placed in a single-perfusion chamber where the slices were in direct contact with the ceramic of the TEC1 device. Briefly, slices were kept in modified aCSF with 120 mM NaCl, 2.5 mM KCl, 1 mM NaH_2_PO_4_, 26 mM NaHCO_3_, 1 mM MgCl_2_, 1.5 mM CaCl_2_, 10 mM D-glucose (~ 300 mmol/kg, pH 7.4). Picrotoxin stimulation was performed with a flow rate of 10 ml/min. After 10 min of imaging Ca^2+^ activity in the baseline to confirm no spontaneous events, 2.5 μM of picrotoxin or DMSO as control was perfused to the slice. During the last 10 min of incubation, intracellular Ca^2+^ was monitored to confirm presence or absence of SLE activity before heat denaturation.

### Histochemical detection of LDH and GAPDH activity

The enzyme activity assays were performed as previously published^60,61^. Briefly, the frozen or heat-frozen cryosections were defrosted for one minute at room temperature and LDH or GAPDH specific assay medium was applied. The LDH reaction was carried out in presence of 150 mM sodium lactate, 3 mM NAD^+^, 0.45 mM methoxyphenanzine methosulfate, 5 mM sodium azide and 5 mM nitroblue tetrazolium chloride in 0.1 M Tris-Maleate buffer pH 7.5. The GAPDH reaction was carried out in presence of 2.5 mM glyceraldehyde-3-phosphate, 3 mM NAD^+^, 0.45 mM methoxyphenanzine methosulfate, 5 mM sodium azide and 5 mM nitroblue tetrazolium chloride in 0.1 M Tris-Maleate buffer pH 7.5. Negative control reactions were performed in presence of 200 mM sodium oxamate or 40 mM iodoacetamine for the LDH and GAPDH reactions, respectively. Enzyme reactions were incubated at room temperature for 5 min. Slides were washed three times in warm PBS and stained for DAPI. After two additional washes, the sections were mounted in Mowiol and imaged on a Zeiss Observer.Z1 with a Plan-Apochromat 20x/0.8 M27 objective and a Zeiss AxioCam MRm camera.

### Immunofluorescence

To visualize the cellular architecture within the acute slices, consecutive tissue sections were used for MSI and immunohistochemistry to identify neurons (nuclear Neun staining) and astrocytes (GFAP staining) in the DG. Acute slices were embedded in 2% CMC, rapidly frozen, and cryosectioned to 10 μm onto superfrost glass slides. For the immunofluorescence, the tissue was post-fixed with 4% PFA for 5 minutes, washed with PBS and blocked and permeabilized for 30 minutes with 3% bovine serum albumin containing 0.01% Tween 20. Following another PBS wash, tissue was incubated overnight at 4°C with conjugated primary antibodies (GFAP-647 abcam ab194325, NeuN-488 EMD Millipore MAB 377X at a concentration of 1:50). Tissue was again washed in PBS and then incubated with DAPI for 5 minutes. After a final PBS wash, slides were mounted with FluorSave and dried overnight in the dark. Images were acquired on a Zeiss Observer.Z1 with a Plan-Apochromat 20x/0.8 M27 objective using Zeiss filter sets 49 (λex:365; λem:445/50), 38(λex:470/40; λem:525/50), and 50(λex:640/30; λem:690/50), and a Zeiss AxioCam MRm camera. The quantification of the area that is covered by the stainings was perfumed using ImageJ. The ROI of the DG was first drawn on the DAPI image and then duplicated onto the NeuN and GFAP image.

### Fast thermal preservation

A thermal assembly using two thermoelectric (Peltier) modules was used to perform the flash heat and flash freeze of each brain slice sample intended for MSI analysis. Details of the construction are illustrated in **Extended Data** Fig. 1a. A 3D-printed chamber-top seals to the top ceramic of the TEC1 device with a 6.0 mm silicone O-ring, creating a physiological chamber of approximately 5.5-mm diameter and 2-mm height. The chamber is perfused continuously during the physiological phase of the experiment; it is then drained rapidly and flipped away from TEC1 (on a hinge) before the flash heat / flash freeze program is initiated.

The high heat-flux TEC1 device is used to perform the rapid temperature changes, and the attached heatsink disk is temperature-controlled to enable those changes. During the physiological experiment, the temperature is controlled by the pre-heated perfusion solution and then adjusted with an inline heater. For fast heating, the perfusion is stopped, and remaining liquid is removed by vacuum suction. The chamber top is lifted and 10 μl of 1% carboxymethyl cellulose (CMC) solution is applied to the slice to preserve tissue integrity during rapid heating. The slice is then immediately heated by applying a pre-determined voltage sequence to TEC1 to pump heat from the heatsink to the top surface. This produces a rapid jump of temperature in the sample to 80°C for 2 seconds, as monitored by the Optris Xi 80 thermal camera (12° lens, Optris Infrared Sensing), using ConnectSDK software.

After the flash heat step, it takes 1.5–2 min to prepare the device for flash freezing. The heatsink is cooled to a temperature of −25 to −35°C using a voltage applied to TEC2. The pumped heat is carried away from the bottom surface of TEC2 by the liquid CPU cooler (PF240-ARGB, SilverStone Technology). During this time, the sample is maintained above freezing (3–6°C) by application of a voltage to TEC1 (calculated based on the thermocouple-monitored temperature of the heatsink and monitored by the thermal camera).

Once the heatsink is cold, the flash freezing step is initiated by rapid reversal of TEC1 from heating to cooling mode. The cooling voltage is applied briefly (~ 300 ms) to rapidly freeze the sample (but avoid injecting too much heat into the heatsink), as monitored by the thermal camera; the sample then stabilizes at the heatsink temperature. At this point, the brain slice sample has been frozen to the top ceramic of TEC1. The TEC1/heatsink assembly (the “A-assembly”) with the frozen sample is placed in a polystyrene tube and kept frozen at −80°C. It is later mounted to a cryostat-chuck adapter (using a tapped hole in the bottom of the heatsink), and the sample is cryosectioned for MALDI MS-imaging.

### Thermal device construction

The thermal assembly (**Extended Data** Fig. 1a) consists of a removable component (A-assembly) and a base assembly. One A-assembly is used for each slice in an experiment and is then stored frozen at −80°C until the sample is thin sectioned; it is cleaned and reused for another experiment. Only a single base assembly is needed.

Each A-assembly consists of (parts listed from top to bottom): Thermoelectric module (TEC1) 1MDL06–050-03AN05 (TEC Microsystems GmbH); Thermal interface film FTS1010 (Toyo Ink) secures TEC1 to the heatsink, by heating in a laboratory oven to 140°C with 0.5–1 MPa of pressure, applied using a weight; Heatsink disk (0.5” diameter × 5 mm) made from tungsten alloy MT-17C (Midwest Tungsten Service; 90% Tungsten, 6% Nickel, 4% Copper). A small hole is drilled in the side to accept a thermocouple for temperature monitoring, and the bottom is drilled and tapped for mounting on top of a cryostat chuck adapter.

The base assembly consists of (parts listed from top to bottom): a graphite thermal interface film (Panasonic EYG-S0909ZLX2); thermoelectric module (TEC2) MS3–119-20–15-00-W8 (Laird Thermal Systems); thermal paste compound Arctic MX-4 (Arctic Cooling); liquid CPU cooler: PF240-ARGB (SilverStone Technology). The base assembly also has a closed cell foam insulator that surrounds the top of TEC2 and the heatsink.

Temperature monitoring and control is performed by a MATLAB program and the following devices: Thermal camera: Xi 80, 12° lens (Optris Infrared Sensing) with images acquired using ConnectSDK software; thermocouple thermometer type T monitored by a TC-08 USB thermocouple data logger (Pico Technology); thermoelectric controller/driver DX5100 OEM5-U (TEC Microsystems GmbH).

### Sample preparation for MSI

Once frozen onto the thermal device, the slice was kept frozen until sectioning. To section, the device was mounted to a custom-made adapter, which fits by screw to the bottom of the heatsink and mounts to a chuck for cryosectioning. The first ~ 50 μm of the slice were usually discarded to get the slice on plane and the tissue was sectioned to 10 μm thickness on a cryostat (Microm HM 550, Thermo Scientific), and thaw-mounted onto indium tin oxide (ITO)-coated glass slides (MALDI IntelliSlides, Bruker Daltonics). If not used on the same day, slides were stored at −80°C before matrix application.

To acquire MSI data, the slides were scanned in a flatbed scanner (Epson Perfection 4490 Photo) at a resolution of 2400 dpi to acquire an optical image and were desiccated until being sprayed with matrix. To make matrix, 39.5 mg 1,5-diaminonaphthalene (DAN) was dissolved in 500 μL 1M HCl with 4 mL HPLC grade H_2_O then vortexed until well mixed and sonicated before adding 4.5 ml 100% HPLC grade EtOH.^15^N-labeled compounds were added to the matrix prior to spraying to calibrate the instrument and could then be used for calibration and as reference points during data analysis. The compounds used were 100 μM ^15^N_1_-glutamate ([M-H]: 147.0418), 10 μM ^15^N_5_-AMP ([M-H]: 351.044), and 1 μM ^15^N_5_-ATP ([M-H]: 510.9692). For the small molecule method, standards were spotted onto the slide to calibrate for this low mass region. The standards used included 100 μM pyruvate ([M-H]: 87.0088), 100 μM lactate ([M-H]: 89.0244), ^15^N_1_-glutamate ([M-H]: 147.0418), and 10 μM ^15^N_5_-AMP ([M-H]: 351.044).

To coat the slide, matrix was loaded into an HTX TM-Sprayer (HTX Technologies) and applied to the slide with a flow rate of 0.09 mL/min, spray nozzle velocity of 1200 mm/min, spray nozzle temperature of 75°C, nitrogen gas pressure of 10 psi, with 4 passes at a spacing of 2 mm. The coated ITO slide was then secured into a Bruker slide adapter and inserted into the mass spectrometer.

### Heat treatment of brain and yeast extracts

To test the effect of heating on metabolite levels, brain tissue was harvested and immediately dropped into 1 mL ice cold 80% MeOH/ 20% water + formic acid. On ice, the tissue was first cut into pieces and then homogenized by sonication, followed by addition of 1 mL chloroform. After vigorous vortexing, the homogenates were centrifuged at 14.000 rpm at 4°C for 10 min and the polar top layer was transferred to a new tube, desiccated in a vacuum concentrator and stored at −80°C. The metabolite pellets of the brain as well as commercially available yeast extract (IROA TruQuant) were taken up in 50% MeOH/ water and 1 μl was spotted onto ITO-coated glass slides and dried in a desiccator. The ITO slide with the extracts was placed on a ~ 90°C hot plate for 15 seconds, while the temperature on top of the slide was being monitored with an infrared thermometer. After the slide cooled down to room temperature, the same brain and yeast extracts were added to the ITO slide and the slides were prepared for MALDI imaging.

### MALDI imaging

Mass spectra were acquired on a timsTOF fleX (Bruker Daltonics, Billerica, MA) in negative ion mode. Using FlexImaging 5.1 software, hippocampal sections were outlined as individual ROIs with a pixel size of 20 μm. Most metabolites were acquired in a single imaging run (*m/z* range 50–1000); however, a separate method was developed to target small metabolites not identified well by the first method, such as hypoxanthine (*m/z* range 50–400). In both methods, each MSI pixel consisted of 1000 laser shots. The laser Smartbeam parameter was set to single with a frequency of 10 kHz. Based on the mass range and selectivity of biomolecules for the two MALDI methods, the instrument parameters were optimized, e.g., quadrupole, ion transfer funnels, collision cell, and focus pre-TOF.

Before each acquisition, the instrument was calibrated by MALDI using the three ^15^N standards spiked into the matrix, as described above. For the small molecule method, a small spot of lactate, pyruvate, and ^15^N_1_-glutamate was dried onto the ITO slide before matrix application, and these three ions were used for calibration of the lower *m/z* range.

### Multiple reaction monitoring

To spectrally resolve the isobaric species of ribose-1-phosphate from other pentose-phosphates, multiple reaction monitoring (MRM) on the timsTOF fleX was implemented scanning over the range of *m/z* 100–500. Using the ESI source, an infusion of each isobaric species was sprayed separately to determine unique fragmentation peaks. The R1P ESI solution was also used to optimize the MRM settings by adjusting the ion transfer funnels, quadrupole, collision cell, and focus pre-TOF parameters. Collision-induced dissociation (CID) was used to isolate the *m/z* 229.01 with a 3 Da width and a collision energy of 18 eV for R1P. The precursor to product ion transition 299.01◊211.00 corresponds to [C_5_H_7_O_7_P-H]^−^ and [C_5_H_11_O_9_P-H]^−^, respectively (**Extended Data** Fig. 3). A MALDI MSI method was then adapted with these instrumental parameters and additional MALDI settings mentioned above. Briefly, DAN-HCl matrix was used, a 20 × 20 μm^2^ pixel size consisting of 1,000 laser shots, and the repetition rate of the laser was 10 kHz.

### MALDI imaging data analysis

All imaging files were opened in SCiLS Lab software (Bruker Daltonics, Billerica, MA) and regions of interest were drawn around the dentate gyrus (DG) in each sample. The ion peak at *m/z* 241.011 was used to identify the dentate granule cells, and the region was conservatively drawn within this border. Total Ion Current (TIC) normalization was performed on all data. An overview spectrum of each region was then exported to a csv file containing a list of ion intensities at all peaks.

This peak list was then imported to the free analysis software, mMass 5.5.0. Peak picking was generally performed with a 0.05% relative intensity threshold (or 0.01% for the small *m/z* method), but adjusted per run if necessary. The resulting peak list was matched against an in-house library, and each list of annotations was adapted according to the spiked ^15^N compounds. The resulting list of annotated metabolites was then exported to an Analysis Report containing the ion intensities at annotated *m/z* peaks. Occasionally, manual peak picking was necessary e.g. in response to inhibitors. An unlabeled tissue sample was included in each ^13^C labeling experiments and labeled metabolites present in that tissue were excluded from analysis.

### Peak assignment and verification

Peak annotations were based on tandem-MS from tissue section, accurate mass measurements, and ion mobility measurements (**Supplementary Data Table 1**). The timsTOF operating in negative ion mode was implemented for tandem-MS measurements. The precursor mass was selected for each metabolite based on MSI data within a 3–10 ppm mass error. Mass spectra were collected by rastering randomly over a 200 μm region and averaging 10 scans, of which each consisted of 1,000 laser shots. The precursor *m/z* isolation window had a range of 0.1–3, which means that in addition to the predicted/standard fragment peaks, some contaminating peaks from nearby compounds are expected. Collision energies were ramped between 7–55 eV, and the spectra were compared to reference spectra from the Metlin or HMDB^62^ databases fragmentation and/or to experimental spectra from purified standards. Fragmentation spectra with hypothetical identities of the fragments are shown in **Supplementary Data Table 1**.

For calculation of cosine scores to evaluate the match between tissue MS-MS spectra and either experimental spectra for purified standards or predicted spectra from a database, the MS-MS spectra were peak-picked (SNR > 5) and binned (50 ppm or 200 ppm). Experimental and standard MS-MS spectra were compared using the cosine similarity^63^. When computing the “standard-specific cosine”, we only considered the peaks present in the standard MS-MS spectrum.

Accurate mass measurements were performed on the 15 Tesla SolariX XR FTICR MS (Bruker Daltonics, Billerica, MA) by imaging serial tissue sections in negative ion mode. A defined pixel size consisted of 50 μm and 200 laser shots, covering from *m/z* 46–3000. To bias the acquisition to improve the sensitivity of small molecules, a continuous accumulation of selected ions (CASI) window was set, Q1 mass was defined at *m/z* 150 with an isolation window of 200 Da. The ion distributions were then compared from the timsTOF and FT-ICR MSI runs. A mass error threshold of < 0.5 ppm using the FT-ICR was used for peak annotation. **Supplementary Data Table 1** shows the search results in PubChem for all chemical formulae matching the accurate mass within 1 ppm, and in each case, the single chemical formula with matches in the HMDB, as well as the names of those matches.

Ion mobility was measured using the trapped ion mobility separation of the timsTOF instrument; for some smaller compounds, the drift tube separation in an Agilent 6560 ion mobility Q-TOF instrument was used (detailed methods below. Ion mobilograms in **Supplementary Data Table 1** plot the ion counts for the target *m/z* peak and the major isotopologues (for ^13^C labeling) as a function of inverse ion mobility (1/*K*_*0*_). These mobilities were compared to standard compounds run in the same session, or to predicted 1/*K*_*0*_ values based on predicted CCS values from the CCSbase database^64^, using the calculation described by Gabelica *et al*.^65^. The table describes the match between measured and standard 1/*K*_*0*_, and the mobilograms show that in almost all cases, the major isotopologues have a mobility matched to the M + 0 species. The exceptions are the 6-phosphogluconate M + 6 and the inosine M + 5 peaks, which sometimes showed contaminants with different mobility; these peaks were therefore omitted from our analysis.

### Calculation of average carbon atom labeling and natural abundance isotope correction

The average carbon atom labeling (atom%) was calculated as previously published^10,11,66,67^ according to the following formula, where f[M+k] is the fractional abundance of the M+k isotopologue

$$atom\%=100\sum_{k=0}^{N}\;kf[M+k]/N$$

For the detailed isotopologue analyses in Fig. 4e–f and **Extended Fig. 4e - g**, a matrix method similar to that described by Millard *et al*.^68^ was used to correct the apparent label fraction for the natural abundance of ^13^C. Because we did not reliably detect every single isotopologue species, for each individual replicate, the values for the reliably detected species were used together with the correction matrix to calculate a non-negative least-squares estimate of the label fraction.

### Agilent Drift Tube Ion Mobility Liquid Chromatography QToF Mass Spectrometry

Analysis for a few compounds with smaller molecular mass was performed on an Agilent 6560 drift tube ion mobility liquid chromatography (LC) coupled to a QToF (Quadrupole Time of Flight) mass spectrometer (LC-MS) (Santa Clara, CA). Both standard and tissue samples were prepared using published procedure ^69,70^ and analyzed using a Waters Atlantis Premier BEH Z-HILIC (Hydrophilic Interaction Chromatography) 1.7 μm (2.1 × 100 mm) column with a flow rate of 0.3 mL/min. The mobile phase consisted of a mixture of 10 mM ammonium carbonate, pH 9.0 (solvent A), and acetonitrile (solvent B). Agilent MassHunter (Santa Clara, CA) software was used for system control, calibrating both drift tube ion mobility and QToF MS prior to the sample run. All the drift tube and QToF MS parameters were kept constant between standards and the tissue samples. Solvents A and B were combined in a gradient: 0–0.5 min: 95% A; 0.5–12 min: 50% A; 12–17 min: 30% A; 17.1–20 min: return to initial conditions. Each sample was injected with 10 μL and the MS was operated in negative ion mode. The chromatographic window containing metabolites of interest was summed to extract both the mass spectra and mobilogram. A tuning mix (Agilent) was used to create a linear function for drift tube calibration, excluding ions that exhibited surfing mode propagation. Collisional cross section (CCS) values were calculated from the fundamental low-field ion mobility Mason-Schamp Eq. 7^1^. The drift time and CCS were calculated for the metabolite of interest for both standards and tissue samples using the Agilent IM-MS Browser software.

### Quantification and statistical analysis

The statistical details of the experiments are provided in the figure legends. In general, the plots show mean ± standard error of the mean (SEM). All manipulations (e.g., KCl stimulation or drug treatments) were done on parallel samples (slices) from the same mouse (i.e., each replicate consisted of a matched control and experimental slice from the same mouse). Unless otherwise stated, peak intensities were normalized to the untreated control and relative calculations were normalized per MSI run. Hypothesis testing for significant differences produced by an experimental manipulation was done by two-tailed, paired Student’s t-tests as implemented in the GraphPad Prism software. Significance is indicated as p < 0.001 (***), p < 0.01 (**), or p < 0.05 (*).

## Data Availability

Key MSI data are deposited at the NIH Common Fund’s National Metabolomics Data Repository (NMDR) Metabolomics Workbench (Study ID ST002699; http://dx.doi.org/10.21228/M8Q716)^72^.

## Abbreviations

2DG: 2-deoxy-D-glucose
8MDP: 2,2’,2’’,2’’’-[[4,8-Bis(hexahydro-1(2H)-azocinyl)pyrimido[5,4-d]pyrimidine-2,6-diyl]dinitrilo]tetrakisethanol
aCSF: artificial cerebrospinal fluid
ADA: adenosine deaminase
AMPD: AMP deaminase
BPG: bisphosphoglycerate
cpd3: (S)-6-(4-(((1-(isoquinolin-8-yl)ethyl)amino)methyl)phenyl)-nicotinic acid
DGC: dentate granule cell
DHAP: dihydroxyacetone phosphate
E4P: erythrose-4-phosphate
MethADP: Adenosine 5’-(α,β-methylene)diphosphate
Foro: forodesine
G6PD: glucose 6-phosphate dehydrogenase
G6PDi: glucose 6-phosphate dehydrogenase inhibitor (4-([5-oxo-6H,7H,8H,9H-cyclohepta[d]pyrimidin-2-yl]amino)thiophene-2-carbonitrile)
GAP: glyceraldehyde-3-phosphate
HPRT: hypoxanthine-guanine phosphoribosyltransferase
IMP: inosine monophosphate
MALDI: matrix assisted laser desorption ionization
MRM: multiple reaction monitoring (MRM)
MS: mass spectrometry
MSI: mass spectrometry imaging
oxPPP: oxidative branch of the PPP
non-oxPPP: non-oxidative branch of the PPP
6PG: 6-phosphogluconate
PCreatine: Phosphocreatine
pentoseP: pentose phosphate
PEP: phosphor-*enol*-pyruvate
PG: phosphoglycerate
PNP: purine nucleotide phosphorylase
PPP: pentose phosphate pathway
R1P: ribose 1-phosphate
R5P: ribose 5-phosphate
Ri5P: ribulose 5-phosphate
ROI: region of interest
S7P: sedoheptulose-phosphate
X5P: xylulose 5-phosphate

## References

[1] Shin T. H. Metabolome Changes in Cerebral Ischemia. Cells 9, E1630 (2020). 10.3390/cells9071630 PMC7407387 32645907

[2] Puig B., Brenna S. & Magnus T. Molecular Communication of a Dying Neuron in Stroke. Int J Mol Sci 19, E2834 (2018). 10.3390/ijms19092834 PMC6164443 30235837

[3] Taoufik E. & Probert L. Ischemic neuronal damage. Curr Pharm Des 14, 3565–3573 (2008). 19075733 10.2174/138161208786848748

[4] Stopka S. A. Spatially resolved characterization of tissue metabolic compartments in fasted and high-fat diet livers. PLoS One 17, e0261803 (2022). 36067168 10.1371/journal.pone.0261803 PMC9447892

[5] Cohen L. H. & Gusev A. I. Small molecule analysis by MALDI mass spectrometry. Anal Bioanal Chem 373, 571–586 (2002). 12219737 10.1007/s00216-002-1321-z

[6] Tsai Y.-H., Garrett T. J., Carter C. S. & Yost R. A. Metabolomic Analysis of Oxidative and Glycolytic Skeletal Muscles by Matrix-Assisted Laser Desorption/IonizationMass Spectrometric Imaging (MALDI MSI). J Am Soc Mass Spectrom 26, 915–923 (2015). 25893271 10.1007/s13361-015-1133-y PMC4553944

[7] Sugiura Y. Visualization of in vivo metabolic flows reveals accelerated utilization of glucose and lactate in penumbra of ischemic heart. Sci Rep 6, 32361 (2016). 27581923 10.1038/srep32361 PMC5007669

[8] Kleinridders A. Regional differences in brain glucose metabolism determined by imaging mass spectrometry. Molecular Metabolism 12, 113–121 (2018). 29681509 10.1016/j.molmet.2018.03.013 PMC6001904

[9] Wang Z. Spatial-resolved metabolomics reveals tissue-specific metabolic reprogramming in diabetic nephropathy by using mass spectrometry imaging. Acta Pharm Sin B 11, 3665–3677 (2021). 34900545 10.1016/j.apsb.2021.05.013 PMC8642449

[10] Wang L. Spatially resolved isotope tracing reveals tissue metabolic activity. Nat Methods 19, 223–230 (2022). 35132243 10.1038/s41592-021-01378-y PMC10926149

[11] Wang G. Analyzing cell-type-specific dynamics of metabolism in kidney repair. Nat Metab 4, 1109–1118 (2022). 36008550 10.1038/s42255-022-00615-8 PMC9499864

[12] Dienel G. A. Stop the rot. Enzyme inactivation at brain harvest prevents artifacts: A guide for preservation of the in vivo concentrations of brain constituents. J Neurochem 158, 1007–1031 (2021). 33636013 10.1111/jnc.15293

[13] Lu W. Metabolite Measurement: Pitfalls to Avoid and Practices to Follow. Annu Rev Biochem 86, 277–304 (2017). 28654323 10.1146/annurev-biochem-061516-044952 PMC5734093

[14] Blatherwick E. Q., Svensson C. I., Frenguelli B. G. & Scrivens J. H. Localisation of adenine nucleotides in heat-stabilised mouse brains using ion mobility enabled MALDI imaging. International Journal of Mass Spectrometry 345–347, 19–27 (2013).

[15] Fox P. T., Raichle M. E., Mintun M. A. & Dence C. Nonoxidative glucose consumption during focal physiologic neural activity. Science 241, 462–464 (1988). 3260686 10.1126/science.3260686

[16] Madsen P. L., Cruz N. F., Sokoloff L. & Dienel G. A. Cerebral oxygen/glucose ratio is low during sensory stimulation and rises above normal during recovery: excess glucose consumption during stimulation is not accounted for by lactate efflux from or accumulation in brain tissue. J. Cereb. Blood Flow Metab. 19, 393–400 (1999). 10197509 10.1097/00004647-199904000-00005

[17] Bak L. K. Neuronal glucose but not lactate utilization is positively correlated with NMDA-induced neurotransmission and fluctuations in cytosolic Ca ^2+^ levels. Journal of Neurochemistry 109, 87–93 (2009). 19393013 10.1111/j.1471-4159.2009.05943.x

[18] Hyder F., Fulbright R. K., Shulman R. G. & Rothman D. L. Glutamatergic Function in the Resting Awake Human Brain is Supported by Uniformly High Oxidative Energy. J Cereb Blood Flow Metab 33, 339–347 (2013). 23299240 10.1038/jcbfm.2012.207 PMC3587823

[19] Díaz-García C. M. Neuronal stimulation triggers neuronal glycolysis and not lactate uptake. Cell Metab. 26, 361–374.e4 (2017). 28768175 10.1016/j.cmet.2017.06.021 PMC5559896

[20] Ghergurovich J. M. A small molecule G6PD inhibitor reveals immune dependence on pentose phosphate pathway. Nat Chem Biol 16, 731–739 (2020). 32393898 10.1038/s41589-020-0533-x PMC7311271

[21] Tozzi M. G., Camici M., Mascia L., Sgarrella F. & Ipata P. L. Pentose phosphates in nucleoside interconversion and catabolism. FEBS J 273, 1089–1101 (2006). 16519676 10.1111/j.1742-4658.2006.05155.x

[22] Ipata P. L., Camici M., Micheli V. & Tozz M. G. Metabolic network of nucleosides in the brain. Curr Top Med Chem 11, 909–922 (2011). 21401502 10.2174/156802611795347555

[23] Ipata P. L. & Balestri F. The functional logic of cytosolic 5’-nucleotidases. Curr Med Chem 20, 4205–4216 (2013). 23992316 10.2174/0929867311320340002

[24] Zhang Y. An RNA-sequencing transcriptome and splicing database of glia, neurons, and vascular cells of the cerebral cortex. J Neurosci 34, 11929–11947 (2014). 25186741 10.1523/JNEUROSCI.1860-14.2014 PMC4152602

[25] Pastor-Anglada M. & Pérez-Torras S. Emerging Roles of Nucleoside Transporters. Front Pharmacol 9, 606 (2018). 29928232 10.3389/fphar.2018.00606 PMC5997781

[26] Lin W. & Buolamwini J. K. Synthesis, flow cytometric evaluation, and identification of highly potent dipyridamole analogues as equilibrative nucleoside transporter 1 inhibitors. J Med Chem 50, 3906–3920 (2007). 17636949 10.1021/jm070311l PMC2536492

[27] Wu Z. A GRAB sensor reveals activity-dependent non-vesicular somatodendritic adenosine release. 2020.05.04.075564 Preprint at 10.1101/2020.05.04.075564 (2020).

[28] Admyre T. Inhibition of AMP deaminase activity does not improve glucose control in rodent models of insulin resistance or diabetes. Chem Biol 21, 1486–1496 (2014). 25459661 10.1016/j.chembiol.2014.09.011

[29] Li G., Nakagome I., Hirono S., Itoh T. & Fujiwara R. Inhibition of adenosine deaminase (ADA)-mediated metabolism of cordycepin by natural substances. Pharmacol Res Perspect 3, e00121 (2015). 26038697 10.1002/prp2.121 PMC4448975

[30] Mohlin C., Säve S., Nilsson M. & Persson K. Studies of the extracellular ATP-adenosine pathway in human urinary tract epithelial cells. Pharmacology 84, 196–202 (2009). 19729987 10.1159/000235908

[31] Martínez-François J. R. BAD and KATP channels regulate neuron excitability and epileptiform activity. Elife 7, (2018). 10.7554/eLife.32721 PMC5785210 29368690

[32] Barry J. A., Groseclose M. R. & Castellino S. Quantification and assessment of detection capability in imaging mass spectrometry using a revised mimetic tissue model. Bioanalysis 11, 1099–1116 (2019). 31251106 10.4155/bio-2019-0035

[33] Källback P. Cross-validated Matrix-Assisted Laser Desorption/Ionization Mass Spectrometry Imaging Quantitation Protocol for a Pharmaceutical Drug and Its Drug-Target Effects in the Brain Using Time-of-Flight and Fourier Transform Ion Cyclotron Resonance Analyzers. Anal Chem 92, 14676–14684 (2020). 33086792 10.1021/acs.analchem.0c03203 PMC7660593

[34] Woods A. S. Lipid/peptide/nucleotide separation with MALDI-ion mobility-TOF MS. Anal Chem 76, 2187–2195 (2004). 15080727 10.1021/ac035376k

[35] Fernandez-Lima F., Kaplan D. A., Suetering J. & Park M. A. Gas-phase separation using a trapped ion mobility spectrometer. Int. J. Ion Mobil. Spec. 14, 93–98 (2011). 10.1007/s12127-011-0067-8 PMC3807855 24163587

[36] Tian H. Multi-modal mass spectrometry imaging reveals single-cell metabolic states in mammalian liver. 2022.09.26.508878 Preprint at 10.1101/2022.09.26.508878 (2022).

[37] Harriott A. M., Takizawa T., Chung D. Y. & Chen S.-P. Spreading depression as a preclinical model of migraine. J Headache Pain 20, 45 (2019). 31046659 10.1186/s10194-019-1001-4 PMC6734429

[38] Charles A. C. & Baca S. M. Cortical spreading depression and migraine. Nat Rev Neurol 9, 637–644 (2013). 24042483 10.1038/nrneurol.2013.192

[39] Aiba I. & Noebels J. L. Spreading depolarization in the brainstem mediates sudden cardiorespiratory arrest in mouse SUDEP models. Sci Transl Med 7, 282ra46 (2015). 10.1126/scitranslmed.aaa4050 PMC4852131 25855492

[40] Gupta S. KL1 Domain of Longevity Factor Klotho Mimics the Metabolome of Cognitive Stimulation and Enhances Cognition in Young and Aging Mice. J Neurosci 42, 4016–4025 (2022). 35428698 10.1523/JNEUROSCI.2458-21.2022 PMC9097772

[41] Lewin E. & Bleck V. Electroshock seizures in mice: effect on brain adenosine and its metabolites. Epilepsia 22, 577–581 (1981). 7285883 10.1111/j.1528-1157.1981.tb04129.x

[42] Barsotti C. & Ipata P. L. Metabolic regulation of ATP breakdown and of adenosine production in rat brain extracts. Int J Biochem Cell Biol 36, 2214–2225 (2004). 15313467 10.1016/j.biocel.2004.04.015

[43] Sahlin K. & Broberg S. Adenine nucleotide depletion in human muscle during exercise: causality and significance of AMP deamination. Int J Sports Med 11 Suppl 2, S62–67 (1990). 10.1055/s-2007-1024856 2361781

[44] Idström J. P., Soussi B., Elander A. & Bylund-Fellenius A. C. Purine metabolism after in vivo ischemia and reperfusion in rat skeletal muscle. Am J Physiol 258, H1668–1673 (1990). 2360663 10.1152/ajpheart.1990.258.6.H1668

[45] Gerlach E., Deuticke B. & Dreisbach R.H. Der Nucleotid-Abbau im Herzmuskel bei Sauerstoffmangel und seine mögliche Bedeutung für die Coronardurchblutung. Naturwissenschaften 50, 228–229 (1963). 10.1007/BF00639287.

[46] Carlson J. D. & Fischer A. G. Thyroid purine nucleoside phosphorylase. II. Kinetic model by alternate substrate and inhibition studies. Biochim Biophys Acta 566, 259–265 (1979). 105760 10.1016/0005-2744(79)90029-9

[47] Erion M. D., Stoeckler J. D., Guida W. C., Walter R. L. & Ealick S. E. Purine nucleoside phosphorylase. 2. Catalytic mechanism. Biochemistry 36, 11735–11748 (1997). 9305963 10.1021/bi961970v

[48] Barsotti C., Pesi R., Felice F. & Ipata P. L. The purine nucleoside cycle in cell-free extracts of rat brain: evidence for the occurrence of an inosine and a guanosine cycle with distinct metabolic roles. Cell Mol Life Sci 60, 786–793 (2003). 12785725 10.1007/s00018-003-2371-x PMC11138790

[49] Abt E. R. Purine nucleoside phosphorylase enables dual metabolic checkpoints that prevent T cell immunodeficiency and TLR7-associated autoimmunity. J Clin Invest 132, e160852 (2022). 35653193 10.1172/JCI160852 PMC9374381

[50] Wang T. Inosine is an alternative carbon source for CD8+-T-cell function under glucose restriction. Nat Metab 2, 635–647 (2020). 32694789 10.1038/s42255-020-0219-4 PMC7371628

[51] Markert M. L. Purine nucleoside phosphorylase deficiency. Immunodefic Rev 3, 45–81 (1991). 1931007

[52] Alangari A., Al-Harbi A., Al-Ghonaium A., Santisteban I. & Hershfield M. Purine nucleoside phosphorylase deficiency in two unrelated Saudi patients. Ann Saudi Med 29, 309–312 (2009). 19584574 10.4103/0256-4947.55320 PMC2841460

[53] Toro A. & Grunebaum E. TAT-mediated intracellular delivery of purine nucleoside phosphorylase corrects its deficiency in mice. J Clin Invest 116, 2717–2726 (2006). 16964310 10.1172/JCI25052 PMC1560347

[54] Nascimento F. P., Macedo-Júnior S. J., Lapa-Costa F. R., Cezar-Dos-Santos F. & Santos A. R. S. Inosine as a Tool to Understand and Treat Central Nervous System Disorders: A Neglected Actor? Front Neurosci 15, 703783 (2021). 34504414 10.3389/fnins.2021.703783 PMC8421806

[55] Liu F. Secondary degeneration reduced by inosine after spinal cord injury in rats. Spinal Cord 44, 421–426 (2006). 16317421 10.1038/sj.sc.3101878

[56] Chen P., Goldberg D. E., Kolb B., Lanser M. & Benowitz L. I. Inosine induces axonal rewiring and improves behavioral outcome after stroke. Proc Natl Acad Sci U S A 99, 9031–9036 (2002). 12084941 10.1073/pnas.132076299 PMC124418

[57] Soares Dos Santos Cardoso F., Blanco Martinez A. M. & Martins de Almeida F. Inosine: a novel treatment for sciatic nerve injury. Neural Regen Res 16, 127–128 (2021). 32788466 10.4103/1673-5374.286969 PMC7818882

[58] Chen T.-W. Ultrasensitive fluorescent proteins for imaging neuronal activity. Nature 499, 295–300 (2013). 23868258 10.1038/nature12354 PMC3777791

[59] Díaz-García C. M., Nathwani N., Martínez-François J. R. & Yellen G. Delivery of AAV for Expression of Fluorescent Biosensors in Juvenile Mouse Hippocampus. Bio-protocol 11, e4259–e4259 (2021). 35087918 10.21769/BioProtoc.4259 PMC8720514

[60] Miller A. Exploring Metabolic Configurations of Single Cells within Complex Tissue Microenvironments. Cell Metab 26, 788–800.e6 (2017). 28889950 10.1016/j.cmet.2017.08.014

[61] Van Noorden C. J. & Frederiks W. M. Enzyme histochemistry: a laboratory manual of current methods. vol. 26 (Oxford University Press, 1992).

[62] Wishart D. S. HMDB 5.0: the Human Metabolome Database for 2022. Nucleic Acids Res 50, D622–D631 (2022). 34986597 10.1093/nar/gkab1062 PMC8728138

[63] Bittremieux W. Comparison of Cosine, Modified Cosine, and Neutral Loss Based Spectrum Alignment For Discovery of Structurally Related Molecules. J Am Soc Mass Spectrom 33, 1733–1744 (2022). 35960544 10.1021/jasms.2c00153

[64] Ross D. H., Cho J. H. & Xu L. Breaking Down Structural Diversity for Comprehensive Prediction of Ion-Neutral Collision Cross Sections. Anal. Chem. 92, 4548–4557 (2020). 32096630 10.1021/acs.analchem.9b05772

[65] Gabelica V. Recommendations for reporting ion mobility Mass Spectrometry measurements. Mass Spectrom Rev 38, 291–320 (2019). 30707468 10.1002/mas.21585 PMC6618043

[66] Schwaiger-Haber M. Using mass spectrometry imaging to map fluxes quantitatively in the tumor ecosystem. Nat Commun 14, 2876 (2023). 37208361 10.1038/s41467-023-38403-x PMC10199024

[67] Andersen J. V. Astrocyte metabolism of the medium-chain fatty acids octanoic acid and decanoic acid promotes GABA synthesis in neurons via elevated glutamine supply. Molecular Brain 14, 132 (2021). 34479615 10.1186/s13041-021-00842-2 PMC8414667

[68] Millard P. IsoCor: isotope correction for high-resolution MS labeling experiments. Bioinformatics 35, 4484–4487 (2019). 30903185 10.1093/bioinformatics/btz209

[69] Mackay G. M., Zheng L., van den Broek N. J. F. & Gottlieb E. Analysis of Cell Metabolism Using LC-MS and Isotope Tracers. Methods Enzymol 561, 171–196 (2015). 26358905 10.1016/bs.mie.2015.05.016

[70] Packer M. R. Raf promotes dimerization of the Ras G-domain with increased allosteric connections. Proc Natl Acad Sci U S A 118, e2015648118 (2021). 33653954 10.1073/pnas.2015648118 PMC7958358

[71] Harrison J. A., Kelso C., Pukala T. L. & Beck J. L. Conditions for Analysis of Native Protein Structures Using Uniform Field Drift Tube Ion Mobility Mass Spectrometry and Characterization of Stable Calibrants for TWIM-MS. J Am Soc Mass Spectrom 30, 256–267 (2019). 30324262 10.1007/s13361-018-2074-z

[72] Sud M. Metabolomics Workbench: An international repository for metabolomics data and metadata, metabolite standards, protocols, tutorials and training, and analysis tools. Nucleic Acids Res 44, D463–470 (2016). 26467476 10.1093/nar/gkv1042 PMC4702780

